# Protocol for a systematic review exploring the psychometric properties of self-report health-related quality of life and subjective wellbeing measures used by adolescents with intellectual disabilities

**DOI:** 10.1186/s13643-022-01957-w

**Published:** 2022-05-02

**Authors:** Stephanie Maguire, Jenny Davison, Marian McLaughlin, Victoria Simms

**Affiliations:** Psychology Research Institute, Ulster University, Cromore Road, Coleraine, Northern Ireland BT52 1SA UK

**Keywords:** Health-related quality of life, Subjective wellbeing, Adolescence, Intellectual disability, Self-report, COSMIN, Measures, Psychometric properties

## Abstract

**Background:**

Whilst there are studies that have systematically reviewed the psychometric properties of quality of life measures for children and young people with intellectual disabilities, these narrowly focus on disease or health conditions. The objective of this planned systematic review is therefore to collate, summarise, and critically appraise the psychometric properties of self-report health-related quality of life (HRQoL) and subjective wellbeing measures used by adolescents (aged 11–16) with an intellectual disability.

**Methods:**

We designed and registered a study protocol for a systematic review of studies which explores the psychometric properties of self-report HRQoL and subjective wellbeing measures used by adolescents with intellectual disabilities. Electronic databases including PsycINFO, CINAHL, MEDLINE, and ERIC will be searched using predefined search terms to identify relevant studies. Quantitative and mixed-methods studies, and studies published in peer-reviewed journals or grey literature, will be included. Review papers, editorials, and case studies will be excluded. Eligible studies should identify self-report measures which assess HRQoL and subjective wellbeing among adolescents with intellectual disabilities. The methodological quality of the included studies will be assessed by applying the COSMIN Risk of Bias checklist. The quality of the evidence (i.e. the total body of evidence used for the overall ratings on each psychometric property of an instrument) will be evaluated in accordance with the modified GRADE guidelines.

**Discussion:**

This systematic review will be among the first to systematically explore the psychometric properties of self-report HRQoL and subjective wellbeing measures used by adolescents with intellectual disabilities. By providing evidence-based knowledge about measures being used in HRQoL and subjective wellbeing research amongst this population, and more importantly how reliable and valid these measures are, the most suitable for use will be identified. Our findings will be of potential interest to clinicians, researchers, and service providers who need information about the methodological quality and the characteristics of measures to make informed decisions about the most reliable and valid tool for a specific purpose. The findings from this study will contribute to the knowledge surrounding available and appropriate measures to use for measuring HRQoL and subjective wellbeing of adolescents with intellectual disabilities, which are necessary to inform intervention development and future health policy.

**Systematic review registration:**

The protocol has been registered at the International Prospective Register of Systematic Reviews (PROSPERO). The registration number is CRD42021231697.

**Supplementary Information:**

The online version contains supplementary material available at 10.1186/s13643-022-01957-w.

## Background

Only recently has the study of health-related quality of life (HRQoL) gained scientific interest and has assumed paramount importance in the identification of health risks and populations’ health status [32]. A related concept is that of subjective wellbeing. Despite some commonalities, HRQoL and subjective wellbeing should be treated as separate concepts [61]. According to the [59] can be defined ‘as a multi-dimensional construct shaped by an individuals’ experiences, beliefs, expectations and perceptions of their position in life in the context of their physical, psychological, and social domains of health’ [53].

Wellbeing has long been considered key to the creation and maintenance of healthy and productive societies [17]. Wellbeing is multifaceted [13, 15, 21] and encompasses both objective (e.g. income, education, health) and subjective (e.g. interpersonal relationships, autonomy) aspects of a person’s life [5, 21, 50, 54]. This approach to measuring perceptions and life experiences has been characterised as *subjective wellbeing*. There is no universally agreed definition of subjective wellbeing, and the term is often used interchangeably with life satisfaction, happiness, and quality of life [50, 54]. Subjective wellbeing falls within the hedonic perspective and can be understood as ‘how people feel and function, both on a personal and social level, and how they evaluate their lives as a whole’ [14, 34].

Intellectual disability (ID) is characterised by significant limitations in both intellectual functioning and adaptive behaviour as expressed in conceptual, social, and practical skills. This disability originates during the developmental period, which is defined operationally as before the individual attains age 22 [47]. The severity of ID is classified into four types, based on an intelligence quotient (IQ) test, namely mild, moderate, severe, and profound ID [46].

Whilst there is a wide range of research examining adolescent’s HRQoL and subjective wellbeing, few of these studies include children and young people with ID [2, 11]. Current large-scale global studies of wellbeing such as the WHO Health Behaviour of School-aged Children (HBSC) and Children’s Worlds do not include children with ID. This is of particular concern given that children and adolescents with ID are more likely to experience diminished mental health, chronic conditions, reduced quality of life, lower socioeconomic status, and social exclusion in comparison with their nonintellectually disabled peers [6, 18]. Many HRQoL and wellbeing measures are usually administered by survey and are not accessible to youth with ID. This population may have difficulty understanding the format and complexity of questions and response scales used in questionnaires designed for same-age peers [4, 11], as intellectual impairment is often associated with difficulties in communication [52], working memory [30], and self-insight [27], all of which are required to respond effectively to questionnaires. In 2011, Scott and colleagues carried out a pilot study including adolescents (aged 11–17 years) with ID/special educational needs in the WHO HBSC questionnaire. Using the standard protocol, just over half of the adolescents were able to ‘complete’ the survey questionnaire; however, a high proportion of their responses proved to be uncodable [49].

Adolescents with ID have traditionally been assessed via proxy reports completed by parents, teachers, or carers as it is assumed that these young persons do not have the capacity to self-report on these domains. While some professionals assume that adolescents with ID do not have the ability to self-report on these domains and cannot reliably report their own HRQoL and subjective wellbeing [31, 52], many researchers and clinicians do take the views of adolescents with ID seriously but acknowledge that it is a challenge [63]. As a result, this leaves adolescents with ID excluded from communicating their HRQoL and subjective wellbeing needs and thus heavily dependent on accurate information of the informants [49]. Limitations exist surrounding the robustness of proxy reflection of non-observable internal states (i.e. feelings), particularly in relation to people whose language limitations mean that they have not been able to tell even close proxies what they think [19]. Every individual has a unique perception of his/her HRQoL and subjective wellbeing which is influenced by context, previous experiences, and personal values [42]. This personal perspective can only be obtained through individuals themselves. Therefore, adolescent’s views should, where possible, be sought directly rather than being inferred from proxy reports [62]. Furthermore, recent research has shown that adolescents and parents’ perceptions of HRQoL and subjective wellbeing can differ, and that adolescents can reliably report their HRQoL and subjective wellbeing, provided that the measure is adapted to be appropriate to their age and cognitive functioning [26, 41].

Authors repeatedly note the underrepresentation of young people with ID in health and wellbeing surveys [25, 50]. A report from the Children’s Worlds survey highlights the exclusion of marginalised children, including those in special educational needs settings, and suggests that there is a real need for ways to be found to ‘include these children in research on children’s lives and wellbeing’ ([45]:16). Research has demonstrated that many adolescents with ID do have the capacity to respond to self-report questionnaires [16, 20], and that adaptations such as pictorial representations and limited forced choice options can strengthen their validity [23]. Thus, there has been an emphasis on finding alternative and more accessible ways to enable this population to complete questionnaires independently, reliably, and confidentially [3, 4]. Increasing attention has been paid to the involvement of young people with ID in the design of wellbeing measures and capturing their views on what wellbeing means to them and what is important to their quality of life [25]. For example, Boström et al. [4] developed the Wellbeing in Special Education Questionnaire (WellSEQ) in cooperation with 113 students (aged 12–16) and teachers in special educational needs settings for children with cognitive disabilities [4]. Both students and teachers participated in the process of cooperative inquiry [22] designing and developing the research instruments in three workshops using different methods (brainstorming, sketching, paper-prototyping, focus group, direct observations, and existing system analysis) [3, 4]. The WellSEQ is specifically tailored to measure self-rated mental and ill health, peer relations, and school and family environment in adolescents with mild or moderate intellectual and developmental disability and is administered via an interactive touch-based application for tablet PCs [4]. The standard method for collecting reliable self-reports from persons with ID has consisted of structured interviews (e.g. [24]), but because this is a highly time-consuming procedure, there has been a shift of focus to design questionnaires specifically for this population. As a result, in the past decade, and in line with the trend of stimulating participatory research used with young people with ID, recent studies have attempted to develop and use self-report HRQoL and subjective wellbeing measures for this target group as opposed to typically used proxy measures.

Researchers have started to address the dearth of instruments suitable for measuring HRQoL and subjective wellbeing with adolescents in the ID population. Whilst there are studies (e.g. [8, 28, 40, 53]) that have systematically reviewed the psychometric properties of QoL measures for young people with ID, these narrowly focus on disease or health conditions [11]. Therefore, an up-to-date systematic review is warranted to summarise and appraise the psychometric properties of HRQoL and subjective wellbeing measures focused on adolescents with ID as they are useful in selecting a measure that is fit for purpose [33, 48]. This review will provide evidence-based knowledge regarding what measures are being used in HRQoL and subjective wellbeing research amongst this population and, more importantly, how reliable and valid these measures are. The most suitable HRQoL and subjective wellbeing measures for use amongst adolescents with ID will be identified, and recommendations will be gathered to determine their reliability (i.e. to identify what works). This is urgently needed as clinicians, researchers, and service providers require information about the methodological quality and the characteristics of measures to make informed decisions about the most reliable and valid tool for a specific purpose [29]. It is expected that this systematic review will assist in informing choice when selecting an instrument for the measurement of HRQoL and subjective wellbeing with this population. It will contribute to the knowledge surrounding available and appropriate measures to use for measuring the HRQoL and subjective wellbeing of adolescents with ID, which are necessary to inform intervention development and future health policy.

The aim of this systematic review is to collate, summarise, and critically appraise the psychometric properties of all self-report HRQoL and subjective wellbeing measures used with adolescents (aged 11–16) with ID. The proposed systematic review will answer the following questions:

1. What self-report measures are being/have been used to assess HRQoL and subjective wellbeing of adolescents with ID?
2. What is the methodological quality of these HRQoL and subjective wellbeing measures for use by adolescents with ID?
3. What self-report measures are the most suitable for assessing HRQoL and subjective wellbeing of adolescents with ID?

## Methods

The present protocol has been written according to the PRISMA-P (Preferred Reporting Items for Systematic Reviews and Meta-Analyses) guidelines [35] (see PRISMA-P checklist is provided in Supplemental file 1). This protocol has been registered at the International Prospective Register of Systematic Reviews (PROSPERO) database (registration number: CRD42021231697).

### Search strategy

The search strategy was developed after consultation with Ulster University’s Faculty of Life and Health Sciences senior librarian. The search strategy is broad to include all research articles that use a psychometric instrument to measure HRQoL and subjective wellbeing among adolescents with ID.

A systematic database search was performed using the Psychological Information Database (PsycINFO), Cumulative Index of Nursing and Allied Health Literature (CINAHL), Medical Literature Analysis Retrieval System Online (MEDLINE), and Education Resources Information Center (ERIC). Key terms pertaining to ID, HRQoL and subjective wellbeing, psychometric properties, measures, and adolescence will be used to search for studies for the review (see Supplemental file 2 for the search strategy and full search strings for each of the five key concepts in each database). Studies published from January 2000 to January 2020 and available in the English language will be considered.

### Eligibility criteria

Studies will be selected according to the criteria outlined below.

#### Measures’ construct of interest

Eligible studies should report testing the psychometric properties of instruments designed for use with adolescents (aged 11–16) with ID to assess their HRQoL and subjective wellbeing. HRQoL includes physical health, psychological health, and social health. Subjective wellbeing includes life satisfaction, happiness, and quality of life. Studies designed for adults or older people with ID, and studies that do not assess HRQoL and subjective wellbeing domains, will be excluded.

#### Target population

The study population will include adolescents between the ages of 11 and 16 years as people younger than 11 are usually considered children, and studies that include people over 16 years may include samples with adults. If studies include adolescents with a broader or narrower age range than 11–16-year olds but encompass 11–16-year olds, they will also be included in our review. Participants must have an ID diagnosis, which is a disability characterised by significant limitations in both intellectual functioning and adaptive behaviour as expressed in conceptual, social, and practical skills [47]. Studies will be excluded if it is not clear whether participants have an ID (mild, moderate, severe, or profound) or where they have other conditions (i.e. autism, epilepsy, or physical disabilities) without specifically noting that they also have an ID.

#### Psychometric properties

To ensure standardisation across articles, the COSMIN taxonomy for psychometric properties will be adhered to [38, 39]. According to the taxonomy, psychometric properties cover three quality domains: reliability, validity, and responsiveness. The reliability domain comprises internal consistency, reliability, and measurement error. The validity domain comprises content validity, structural validity, hypotheses testing for construct validity, cross-cultural validity, and criterion validity. The responsiveness domain contains one measurement property, that is responsiveness. Where available, all three quality domains will be assessed in this review.

#### Study designs

Quantitative and mixed-methods studies, and studies published in peer-reviewed journals or grey literature, will be included. Reference lists of all included full texts will be hand searched to identify additional eligible instruments and studies. Websites of any charitable and non-governmental organisations will also be searched for additional reports and papers to identify potential instruments. Both searches for reference lists and websites will be conducted by one reviewer, and any additional identified instruments and studies will be checked by the other reviewer. Review papers, editorials, or case studies will be excluded.

#### Setting

There will be no restrictions by type of setting.

#### Language

We will include studies and instruments developed and published in English. Studies that have not been translated into English will be excluded.

### Data Extraction

Data will be extracted as follows.

#### Literature search and study selection

The titles and abstracts of studies retrieved from the database searches and those from additional sources will be screened independently in Covidence by two review authors (SM and JD) to identify studies that meet the inclusion criteria outlined above. The full text of these potentially eligible studies will be retrieved and independently assessed for eligibility by two review authors. Any disagreement between them over the eligibility of studies will be resolved through discussion with a third review author (MM). The interrater agreement will be assessed by calculating weighted k [9] and interpreted as very good (0.81–1.00), good (0.61–0.80), moderate (0.41–0.60), fair (0.21–0.40), and poor (0.00–0.20) [1].

A standardised form will be used to extract data from included studies in the systematic review. Two independent reviewers will carry out data extraction to reduce bias and errors [51]. Data abstracted will include demographic characteristics, instruments, measurement domains, sample size, sample age, and psychometric properties assessed (see Table 1 in Appendix A for the data extraction form). Reviewers will resolve disagreements by discussion, and an arbitrator (MM) will adjudicate disagreements. We will contact study authors to resolve any uncertainties [7].

### Study quality assessment and data synthesis

#### Evaluation of methodological quality of studies

Studies evaluating the measurement properties of an assessment require high methodological quality with a low-risk bias to guarantee that appropriate conclusions are drawn about the properties of the measure [57]. Thus, it is important to evaluate those methodological qualities [12]. Two review authors will assess the methodological quality by applying the COnsensus-based Standards for the selection of health status Measurement INstruments (COSMIN) Risk of Bias checklist [36, 37]. The COSMIN checklist contains ten boxes with standards for patient-reported outcome measure (PROM) development (box 1) and for nine measurement properties, including content validity (box 2), structural validity (box 3), internal consistency (box 4), cross-cultural validity/measurement invariance (box 5), reliability (box 6), measurement error (box 7), criterion validity (box 8), hypotheses testing for construct validity (box 9), and responsiveness (box 10) [36, 37]. Refer to Table 2 in Appendix B for the definitions of all psychometric properties defined by the COSMIN statement [38, 39]. Only psychometric properties that are assessed in each included study will be completed since not all psychometric properties are assessed in all articles. An overall judgement will be provided on the methodological quality of the studies. We will use a four-point rating system where each standard within a COSMIN box will be rated as 4 = ‘very good’, 3 = ‘adequate’, 2 = ‘doubtful,’ or 1 = ‘inadequate’ to assess the quality of a study on that specific measurement property [36, 37]. The overall rating of each study will be determined by taking the lowest rating of any standard in the box (i.e. ‘the worst score counts’ principle) [58]; however, this makes it difficult to differentiate between subtle psychometric qualities of assessments. Therefore, a revised scoring system will be applied and presented as a percentage: poor (0–25%), fair (25.1–50.0%), good (50.1–75%), and excellent (75.1–100%) [10]. As some COSMIN items only have an option to rate as good or excellent, the total score for each psychometric property will be calculated using the formula detailed below, to accurately capture the quality of psychometric properties [38, 39]:

$$\mathrm{Total}\ \mathrm{score}\ \mathrm{for}\ \mathrm{psychometric}\ \mathrm{property}=\frac{\left(\mathrm{Total}\ \mathrm{score}\ \mathrm{obtained}-\mathrm{minimum}\ \mathrm{score}\ \mathrm{possible}\right)}{\left(\operatorname{Max}\ \mathrm{score}\ \mathrm{possible}-\mathrm{minimum}\ \mathrm{score}\ \mathrm{possible}\right)}\times 100$$

Two review authors will rate the methodological quality independently, and any discrepancies will be resolved by consensus.

#### Evaluation of psychometric properties of instruments

The result of each single study on a psychometric property will be rated against the updated criteria for good measurement properties [56] on which consensus will be achieved by two review authors. Each result will be rated as either sufficient (above the quality criteria threshold: +), insufficient (below the quality criteria threshold: −), or indeterminate (less robust data that do not meet the quality criteria: ?) using the predefined criteria for good psychometric properties [36, 37] (see Table 2 in Appendix C).

The evidence will be summarised, and the quality of the evidence (i.e. the total body of evidence used for the overall ratings on each psychometric property of an instrument) will be graded as high, moderate, low, or very low using the modified Grading of Recommendations, Assessment, Development and Evaluations (GRADE) approach (see [43]). The quality assessment will be done by two reviewers independently, and that consensus among the reviewers is reached, if necessary, with the help of a third reviewer.

#### Selection of instruments

The selection of instruments and recommendation of suitable instruments for future use will be based on a combining overall rating results of each psychometric property (step 2) and the grading results (step 3) [43]. Each instrument will be classified into three recommendation categories [36, 37]: (A) instruments that have potential to be recommended as the most suitable instrument for the construct and population of interest; (B) instruments that may have the potential to be recommended, but further validation studies are needed (i.e. instruments categorized not in A or C); and (C) instruments that should not be recommended. Justifications will be given as to why an instrument is placed in a certain category, and direction will be given on future validation work, if applicable [43].

## Discussion

To the best of our knowledge, this is the first systematic review exploring the psychometric properties of self-report HRQoL and subjective wellbeing measures used by adolescents with ID. By providing evidence-based knowledge about measures being used in health and wellbeing research amongst this population, and more importantly how reliable and valid these measures are, the most suitable for use will be identified. This is crucial as clinicians, researchers, and service providers need information about the methodological quality and the characteristics of measures to make informed decisions about the most reliable and valid tool for a specific purpose. The findings of this study will contribute to the knowledge surrounding available and appropriate measures to use for measuring the HRQoL and subjective wellbeing of adolescents with ID, which are necessary to inform intervention development and future health policy.

One limitation of the systematic review process is the exclusion of studies that are not available in the English language, which may mean that articles identifying HRQoL and subjective wellbeing measures used with adolescents with ID based in non-English-speaking countries and not available in the English language will be excluded. The interpretability and feasibility of measurement tools have also been shown to be important in understanding the overall quality of an instrument [60]. However, as this study utilises the COSMIN criteria which does not provide an assessment of these constructs, they will not be appraised and thus is another limitation of our systematic review.

## Supplementary Information

**Additional file 1:.** PRISMA-P 2015 Checklist.

**Additional file 2.** Search Strategy.

## Data Availability

The studies included in the review will be available from the corresponding author upon request.

## Appendix A

Table 1

**Table 1:** Data extraction form

Author and date	Country	Instrument	Measurement domains	Sample size	Study population	Sample age	Psychometric properties assessed
⠀							

## Appendix B

Table 2

**Table 2:** COSMIN definitions of domains, measurement properties, and aspects of measurement properties [38, 39]

Term			Definition
Domain	Measurement property	Aspect of a measurement property	
Reliability			The degree to which the measurement is free from measurement error
Reliability (extended definition)			The extent to which scores for patients who have not changed are the same for repeated measurement under several conditions, e.g. using different sets of items from the same patient-reported outcome measure (PROM) (internal consistency); over time (test-retest); by different persons on the same occasion (interrater); or by the same persons (i.e. raters or responders) on different occasions (intra-rater)
	Internal consistency		The degree of the interrelatedness among the items
	Reliability		The proportion of the total variance in the measurements which is due to ‘true’^a^ differences between patients
	Measurement error		The systematic and random error of a patient’s score that is not attributed to true changes in the construct to be measured
Validity			The degree to which a PROM measures the construct(s) it purports to measure
	Content validity		The degree to which the content of a PROM is an adequate reflection of the construct to be measured
		Face validity	The degree to which (the items of) a PROM indeed looks as though they are an adequate reflection of the construct to be measured
	Construct validity		The degree to which the scores of a PROM are consistent with hypotheses (for instance, with regard to internal relationships, relationships to scores of other instruments, or differences between relevant groups) based on the assumption that the PROM validly measures the construct to be measured
		Structural validity	The degree to which the scores of a PROM are an adequate reflection of the dimensionality of the construct to be measured
		Hypotheses testing	Idem construct validity
		Cross-cultural validity	The degree to which the performance of the items on a translated or culturally adapted PROM is an adequate reflection of the performance of the items of the original version of the PROM
	Criterion validity		The degree to which the scores of a PROM are an adequate reflection of a ‘gold standard’
Responsiveness			The ability of a PROM to detect change over time in the construct to be measured
		Responsiveness	Idem responsiveness
Interpretability^b^			Interpretability is the degree to which one can assign qualitative meaning — that is, clinical or commonly understood connotations — to a PROM’s quantitative scores or change in scores

^a^The word ‘true’ must be seen in the context of the CTT, which states that any observation is composed of two components — a true score and error associated with the observation. ‘True’ is the average score that would be obtained if the scale was given an infinite number of times. It refers only to the consistency of the score, and not to its accuracy [55] ^b^Interpretability is not considered a measurement property but an important characteristic of a measurement instrument

## Appendix C

Table 3

**Table 3:** Criteria for good psychometric properties adapted from Prinsen et al. [43]

Measurement property	Rating^**a**^	Criteria
Structural validity	+	**CTT** • CFA: CFI or TLI or comparable measure > 0.95 OR RMSEA • < 0.06 OR SRMR < 0.082 **IRT/Rasch** No violation of unidimensionality^c^: CFI or TLI or comparable measure > 0.95 OR RMSEA < 0.06V OR SRMR < 0.08^b^ *AND* No violation of local independence: residual correlations amongthe items after controlling for the dominant factor < 0.20 ORQ3s < 0.37 *AND* no violation of monotonicity: adequate looking graphs OR item scalability > 0.30 *AND* Adequate model fit • IRT: *χ*^2^ >0 .01 • Rasch: infit and outfit mean squares ≥ 0.5 and ≤ 1.5 OR Z-standardized values > −2
	?	CTT: not all information for ‘+’ reported IRT/Rasch: model fit not reported
	–	Criteria for ‘+’ not met
Internal consistency	+	At least low evidence^d^ for sufficient structural validity^e^ AND Cronbach’s alpha(s) ≥ 0.70 for each unidimensional scale or subscale^f^
	?	Criteria for ‘At least low evidence^d^ for sufficient structural validity^e^’ not met
	–	At least low evidence^d^ for sufficient structural validity_5_ AND Cronbach’s alpha(s) < 0.70 for each unidimensional scale or subscale^f^
Reliability	+	ICC or weighted kappa ≥ 0.70
	? –	ICC or weighted kappa not reported ICC or weighted kappa < 0.70
Measurement error	+ ? –	SDC or LoA < MIC^e^ MIC not defined SDC or LoA > MIC^e^
Hypotheses testing for construct validity	+	The result is in accordance with the hypothesis^g^
	?	No hypothesis defined (by the review team)
	–	The result is not in accordance with the hypothesis^g^
Cross-cultural validity/measurement invariance	+	No important differences found between group factors (such as age, gender, language) in multiple group factor analysis OR no important DIF for group factors (McFadden’s *R*^2^ < 0.02)
	?	No multiple group factor analysis OR DIF analysis performed
	–	Important differences between group factors OR DIF was found
Criterion validity	+	Correlation with gold standard ≥ 0.70 OR AUC ≥ 0.70
	?	Not all information for ‘+’ reported
	–	Correlation with gold standard < 0.70 OR AUC < 0.70
Responsiveness	+	The result is in accordance with the hypothesis^g^ OR AUC ≥ 0.70
	?	No hypothesis defined (by the review team)
	–	The result is not in accordance with the hypothesis^g^ OR AUC < 0.70

The criteria are based on, e.g. Terwee et al. [56] and Prinsen et al. [44]. *AUC*, area under the curve; *CFA*, confirmatory factor analysis; *CFI*, comparative fit index; *CTT*, classical test theory; *DIF*, differential item functioning; *ICC*, intraclass correlation coefficient; *IRT*, item response theory; *LoA*, limits of agreement; *MIC*, minimal important change; *RMSEA*, root-mean-square error of approximation; *SEM*, standard error of measurement; *SDC*, smallest detectable change; *SRMR*, standardized root mean residuals; *TLI*, Tucker-Lewis index. ^a^‘+,’ sufficient; ‘–,’ insufficient, ‘ ?,’ indeterminate. ^b^To rate the quality of the summary score, the factor structures should be equal across studies. ^c^Unidimensionality refers to a factor analysis per subscale, while structural validity refers to a factor analysis of a multidimensional patient-reported outcome measure. ^d^As defined by grading the evidence according to the GRADE approach. ^e^This evidence may come from different studies. ^f^The criteria ‘Cronbach alpha < 0.95’ was deleted, as this is relevant in the development phase of a PROM and not when evaluating an existing PROM. ^g^The results of all studies should be taken together, and it should then be decided if 75% of the results are in accordance with the hypotheses

## Abbreviations

HRQoL: Health-related quality of life
ID: Intellectual disability
COSMIN: COnsensus-based Standards for the selection of health status Measurement INstruments
PsycINFO: Psychological information
CINAHL: Cumulative Index of Nursing and Allied Health Literature
MEDLINE: Medical Literature Analysis Retrieval System Online
ERIC: Education Resources Information Center
GRADE: Grading of Recommendations, Assessment, Development and Evaluations
PRISMA-P: Preferred Reporting Items for Systematic Reviews and Meta-Analyses
PROM: Patient-reported outcome measure

## References

[1] Altman DG (1990). Practical Statistics for Medical Research.

[2] Boström P, Broberg M (2018). Protection and restriction: a mixed-methods study of self-reported well-being among youth with intellectual disabilities. J Appl Res Intellect Disabil.

[3] Boström P, Eriksson E (2015). Design for self-reporting psychological health in children with intellectual disabilities. I Proceedings of IDC 2015: The 14th International Conference on Interaction Design and Children (s. 279-282). [2771896].

[4] Boström P, Johnels AJ, Thorson M, Broberg M (2016). Subjective mental health, peer relations, family, and school environment in adolescents with intellectual developmental 482 disorder: a first report of a new questionnaire administered on tablet PCs. J Ment Health Res Intellect Disabil.

[5] Bowling A. Do older and younger people difer in their reported well-being? A National Survey of Adults in Britain, Family Practice. 2011;28:145–55. 10.1093/fampra/cmq082. 10.1093/fampra/cmq082 PMC3062779 21084566

[6] Buckley N, Glasson EJ, Chen W, Epstein A, Leonard H, Skoss R (2020). Prevalence estimates of mental health problems in children and adolescents with intellectual disability: a systematic review and meta-analysis. Aust N Z J Psychiatry.

[7] Busse JW, Ebrahim S, Connell G, Coomes EA, Bruno P, Malik K, et al. Systematic review and network meta-analysis of interventions for fibromyalgia: a protocol. Unknown J. 2013;2(18). 10.1186/2046-4053-2-18. 10.1186/2046-4053-2-18 PMC3610251 23497523

[8] Carlon S, Shields N, Yong K, Gilmore R, Sakzewski L, Boyd R (2010). A systematic review of the psychometric properties of quality-of-life measures for school aged children with cerebral palsy. BMC Pediatr.

[9] Cohen J, Humphreys LH (1968). Weighted kappa: nominal scale agreement provision for scaled disagreement or partial credit. Psychol Bull.

[10] Cordier R, Speyer R, Chen YW, Wilkes-Gillan S, Brown T, Bourke-Taylor H (2015). Evaluating the psychometric quality of social skills measures: a systematic review. PLoS One.

[11] Davidson G, Irvine R, Corman M, Kee F, Kelly B, Leavey G (2017). Measuring the Quality of Life of People with Disabilities and their Families: Scoping Study Final Report.

[12] De Vet HC, Terwee CB, Mokkink LB, Knol DL, De Vet HCW, Terwee CB, Mokkink LB, Knol DL (2011). Systematic reviews of measurement properties. Measurement in Medicine: A practical Guide.

[13] Delle Fave A, Brdar I, Freire T (2011). The eudaimonic and hedonic components of happiness: qualitative and quantitative findings. Soc Indic Res.

[14] Diener E (2009). The science of well-being: the collected works of Ed Diener.

[15] Dodge R, Daly AP, Huyton J, Sanders LD (2012). The challenge of defining wellbeing. Int J Wellbeing.

[16] Douma J, Dekker MC, Verhulst FC, Koot HM (2006). Self-reports on mental health problems of youth with moderate to borderline intellectual disabilities. J Am Acad Child Adolesc Psychiatry.

[17] Durand M (2015). The OECD better life initiative: how’s life? and the measurement of well-being. Rev Income Wealth.

[18] Emerson E (2021). Inequalities and inequities in the health of people with intellectual disabilities. Oxford Research Encyclopedia of Global Public Health.

[19] Emerson E, Felce D, Stancliffe RJ (2013). Issues concerning self-report data and population-based data sets involving people with intellectual disabilities. Intellect Dev Disabil.

[20] Emerson E, Robertson J, Wood J (2005). Emotional and behavioural needs of children and adolescents with intellectual disabilities in an urban conurbation. J Intellect Disabil Res.

[21] Forgeard MJC, Jayawickreme E, Kern M, Seligman MEP (2011). Doing the right thing: measuring wellbeing for public policy. Int J Wellbeing.

[22] Frauenberger C, Good J, Keay-Bright W (2011). Designing technology for children with special needs: bridging perspectives through participatory design. CoDesign.

[23] Hartley SL, MacLean WE (2006). A review of the reliability and validity of Likert-type scales for people with intellectual disability. J Intellect Disabil Res.

[24] Haynes A, Gilmore L, Shochet I, Campbell M, Roberts C (2013). Factor analysis of the self-report version of the strengths and difficulties questionnaire in a sample of children with intellectual disability. Res Dev Disabil.

[25] Hicks S, Newton J, Haynes J, Evans J (2011). Measuring children’s and young people’s well-being.

[26] Ingerski LM, Modi AC, Hood KK, Pai AL, Zeller M, Piazza-Waggoner C (2010). Health-related quality of life across pediatric chronic conditions. J Pediatr.

[27] Jahoda A, Wilson A, Stalker K, Cairney A (2010). Living with stigma and the self-perceptions of people with mild intellectual disabilities. J Soc Issues.

[28] Jardine J, Glinianaia SV, McConachie H, Embleton ND, Rankin J (2014). Self-reported quality of life of young children with conditions from early infancy: a systematic review. Pediatrics.

[29] Kipfer S, Pihet S (2019). Reliability, validity and relevance of needs assessment instruments for informal dementia caregivers: a psychometric systematic review. JBI Database System Rev Implement Rep.

[30] Lifshitz H, Shtein S, Weiss I, Vakil E (2011). Meta-analysis of explicit memory studies in populations with intellectual disability. Eur J Spec Needs Educ.

[31] Longo E, Badia M, Orgaz MB, Gómez-Vela M (2017). Comparing parent and child reports of health-related quality of life and their relationship with leisure participation in children and adolescents with cerebral palsy. Res Dev Disabil.

[32] Marques A, Peralta M, Santos T, Martins J, Gaspar de Matos M (2019). Self-rated health and health-related quality of life are related with adolescents’ healthy lifestyle. Public Health.

[33] Mason SJ, Catto J, Downing A, Bottomley SE, Glaser AW, Wright P (2018). Evaluating patient-reported outcome measures (PROMs) for bladder cancer: a systematic review using the COnsensus-based Standards for the selection of health Measurement INstruments (COSMIN) checklist. BJU Int.

[34] Michaelson J, Mahony S, Schifferes J (2012). Measuring wellbeing: a guide for practitioners.

[35] Moher D, Shamseer L, Clarke M, Ghersi D, Liberati A, Petticrew M (2015). Preferred reporting items for systematic review and meta-analysis protocols (PRISMA-P) 2015 statement. Syst Rev.

[36] Mokkink LB, de Vet H, Prinsen C, Patrick DL, Alonso J, Bouter LM (2018). COSMIN Risk of Bias checklist for systematic reviews of patient-reported outcome measures. Qual Life Res Int J Qual Life Asp Treat Care Rehab.

[37] Mokkink LB, Prinsen CAC, Patrick DL, Alonso J, Bouter LM, de Vet HCW (2018). COSMIN methodology for systematic reviews of patient-reported outcome measures (PROMs)-user manual (version 1.0).

[38] Mokkink LB, Terwee CB, Knol DL, Stratford PW, Alonso J, Patrick DL (2010). The COSMIN checklist for evaluating the methodological quality of studies on measurement properties: a clarification of its content. BMC Med Res Methodol.

[39] Mokkink LB, Terwee CB, Patrick DL, Alonso J, Stratford PW, Knol DL (2010). The COSMIN study reached international consensus on taxonomy, terminology, and definitions of measurement properties for health-related patient-reported outcomes. J Clin Epidemiol.

[40] Morris C, Janssens A, Shilling V, Allard A, Fellows A, Tomlinson R, et al. Meaningful health outcomes for paediatric neurodisability: stakeholder prioritisation and appropriateness of patient reported outcome measures. Health Qual Life Outcomes. 2015;13(87). 10.1186/s12955-015-0284-7. 10.1186/s12955-015-0284-7 PMC4478638 26108625

[41] Morrow AM, Hayen A, Quine S, Scheinberg A, Craig JC (2011). A comparison of doctors’, parents’ and children’s reports of health states and health-related quality of life in children with chronic conditions. Child Care Health Dev.

[42] Noonan RJ, Boddy LM, Fairclough SJ, Knowles ZR (2016). Write, draw, show, and tell: a child-centred dual methodology to explore perceptions of out-of-school physical activity. BMC Public Health.

[43] Prinsen C, Mokkink LB, Bouter LM, Alonso J, Patrick DL, de Vet H (2018). COSMIN guideline for systematic reviews of patient-reported outcome measures. Qual Life Res Int J Qual Life Asp Treat Care Rehab.

[44] Prinsen CA, Vohra S, Rose MR, Boers M, Tugwell P, Clarke M, et al. How to select outcome measurement instruments for outcomes included in a “core outcome set” - a practical guideline. Trials. 2016;17(1). 10.1186/s13063-016-1555-2. 10.1186/s13063-016-1555-2 PMC5020549 27618914

[45] Rees G, Main G (2015). Children’s views on their lives and well-being in 15 countries: A report on the Children’s Worlds Survey, 2013-14.

[46] Schalock RL, Borthwick-Duffy SA, Bradley VJ, Buntinx WH, Coulter DL, Craig EM (2010). Intellectual disability: Definition, classification, and systems of supports.

[47] Schalock RL, Luckasson R, Tassé MJ (2021). Twenty questions and answers regarding the 12th edition of the AAIDD manual: intellectual disability: definition, diagnosis, classification, and systems of supports.

[48] Scholtes VA, Terwee CB, Poolman RW (2011). What makes a measurement instrument valid and reliable?. Injury.

[49] Scott J, Wishart J, Currie C (2011). Including children with intellectual disabilities/special educational needs into national child health surveys: a pilot study. J Appl Res Intellect Disabil.

[50] Selwyn J, Wood M (2015). Measuring Well-Being: A Literature review.

[51] Shamseer L, Moher D, Clarke M, Ghersi D, Liberati A, Petticrew M (2015). Preferred reporting items for systematic review and meta-analysis protocols (PRISMA-P) 2015: elaboration and explanation. BMJ (Clinical research ed).

[52] Shevell M (2008). Global developmental delay and mental retardation or intellectual disability: conceptualization, evaluation, and etiology. Pediatr Clin N Am.

[53] Solans M, Pane S, Estrada MD, Serra-Sutton V, Berra S, Herdman M (2008). Health-related quality of life measurement in children and adolescents: a systematic review of generic and disease-specific instruments. Value Health.

[54] Statham J, Chase E (2010). Childhood Wellbeing: A brief overview.

[55] Streiner DL, Norman GR (2008). Health Measurement Scales: A Practical Guide to Their Development and Use.

[56] Terwee CB, Bot SD, de Boer MR, van der Windt DA, Knol DL, Dekker J (2007). Quality criteria were proposed for measurement properties of health status questionnaires. J Clin Epidemiol.

[57] Terwee CB, Jansma EP, Riphagen II, de Vet HC (2009). Development of a methodological PubMed search filter for finding studies on measurement properties of measurement instruments. Qual Life Res Int J Qual Life Asp Treat Care Rehab.

[58] Terwee CB, Mokkink LB, Knol DL, Ostelo RW, Bouter LM, de Vet HC (2012). Rating the methodological quality in systematic reviews of studies on measurement properties: a scoring system for the COSMIN checklist. Qual Life Res Int J Qual Life Asp Treat Care Rehab.

[59] The World Health Organization Quality of Life Assessment (WHOQOL) (1998). Development and general psychometric properties. Soc Sci Med.

[60] Thornicroft G, Slade M (2000). Are routine outcome measures feasible in mental health?. Qual Health Care.

[61] Upton D, Upton P (2015). Quality of Life and Well-Being. Psychology of Wounds and Wound Care in Clinical Practice.

[62] Upton P, Lawford J, Eiser C. Parent–child agreement across child health-related quality of life instruments: a review of the literature. Qual Life Res. 2008;17(6):895-913. 10.1007/s11136-008-9350-5. 10.1007/s11136-008-9350-5 18521721

[63] White-Koning M, Arnaud C, Bourdet-Loubère S, Bazex H, Colver A, Grandjean H (2005). Subjective quality of life in children with intellectual impairment--how can it be assessed?. Dev Med Child Neurol.

