# Supplementary material for: Protocol for a systematic review exploring the psychometric properties of self-report health-related quality of life and subjective wellbeing measures used by adolescents with intellectual disabilities
Source: Syst Rev. 2022 May 2;11:81. doi: 10.1186/s13643-022-01957-w (PMC9063098; doi:10.1186/s13643-022-01957-w)
Supplement: Supplementary file 2 — Additional file 2. Search Strategy. [file 13643_2022_1957_MOESM2_ESM.docx]

**Search Strategy**

**Database Host:** Ovid

**Database:** PsycINFO

**Date:** 3.2.2022

**Results:** 1,148

**Limits:** English Language; Publication Year: 2000 - 2020

| **Concept 1** | **Concept 2** | **Concept 3** | **Concept 4** | **Concept 5** |
| --- | --- | --- | --- | --- |
| **HRQoL and Subjective Wellbeing** | **Measures** | **Psychometric Properties** | **Adolescence** | **Intellectual Disability** |
| 1. exp "Health Related Quality of Life"/ or exp Adolescent Health/ or exp Mental Health/ or exp Health Outcomes/  2. exp Mental Health/ or exp Life Satisfaction/ or exp Well Being/  3. "quality of life"/ or "quality of life measures"/ or wellbeing/  4.exp Happiness/ or exp Social Support/ or exp social health  5. exp Physical Health/  6. (wellness or wellbeing or well-being or subjective health or subjective wellbeing or objective health or objective wellbeing or hedonic wellbeing or eudaimonic wellbeing or mental wellbeing or psychological wellbeing or psychological health or physical wellbeing)  7. 1 or 2 or 3 or 4 or 5 or 6 | 8. exp questionnaires/  9. exp Surveys/  10.Self-Report/  11.exp Self-Evaluation/  12. exp Measurement/  13. (scale* or indicator* or instrument* or tool* or assessment instrument* or assessment*)  14. 8 or 9 or 10 or 11 or 12 or 13  15. 7 and 14 | 16. exp Psychometrics/  17. (valid* or reliab*)  18. 16 or 17 | 19. exp Early Adolescence/  20. (youth* or young people or teen* or child*)  21. 19 or 20 | 22. exp Cognitive Impairment/ or exp Intellectual Development Disorder/ or exp Disabilities/ or exp Developmental Disabilities/  23. exp Neurodevelopmental Disorders/  24. learning disabilities/ or learning disorders/  25. delayed development/ or developmental disabilities/  26. mental dsiorders/  27. (learning difficult* or learning deficien* or intellectual impair* or intellectual disorder* or intellectual deficien* or handicap* or impair* or subnormal or mental retard* or mental disab* or mental impair* or mental handicap* or mental deficien*)  28. ("cerebral palsy" or "Fragile X syndrome" or "Down syndrome" or "Prader-Willi syndrome" or "Smith-Magenis syndrome" or "Rett syndrome" or "Lesch Nyhan syndrome" or "Angelman Syndrome" or "Cri du Chat Syndrome" or "Cornelia de Lange syndrome" or "Rubinstein-Taybi syndrome" or "DiGeorge syndrome " or "Williams syndrome" or phenylketonuria or "Fetal alcohol spectrum disorder" or "Autis* spectrum disorder" or Autism or "Attention deficit disorder").  29. 22 or 23 or 24 or 25 or 26 or 27 or 28  30. 15 and 18 and 21 and 29  31. limit 30 to (English language and yr="2000-2020" |

**Database Host:** EBSCO

**Database:** CINAHL Complete

**Date:** 3.2.2022

**Results:** 1,897

**Limits:** English Language; Publication Year: 2000 – 2020

| **Concept 1** | **Concept 2** | **Concept 3** | **Concept 4** | **Concept 5** |
| --- | --- | --- | --- | --- |
| **HRQoL and Subjective Wellbeing** | **Measures** | **Psychometric Properties** | **Adolescence** | **Intellectual Disability** |
| S1. (MH "Quality of Life+") OR (MH "Psychological Well-Being")  S2. (MH "Personal Satisfaction+") OR (MH "Wellness")  S3. wellbeing or well-being or subjective health or subjective wellbeing or objective wellbeing or eudaimonic wellbeing or hedonic wellbeing or happiness or self-concept or social support or mental wellbeing or psychological health or health related quality of life or social health  S4. (MH "Mental Health")  S5. S1 OR S2 OR S3 OR S4 | S6. (MH "Self Report+")  S7. (MH "Scales")  S8. measure* or measure* tool* or survey* or questionnaire* or tool* or instrument* or indicator* or health outcome* or assessment* or evaluation*  S9. S6 OR S7 OR S8  S10. S5 AND S9 | S11. ((MH "Reliability and Validity+")  S12. (MH "Psychometrics")  S13. reliab* or valid*  S14. S11 OR S12 OR S13 | S15. (MH "Adolescence+")  S16. Youth* or Young people or Teen* or Child*)  S17. S15 OR S16 | S18. (MH "Intellectual Disability+") OR (MH "Learning Disorders+") OR (MH "Developmental Disabilities") OR (MH "Child, Disabled") OR (MH "Disabled+")  S19. (MH "Mental Retardation, X-Linked+") OR (MH "Mentally Disabled Persons")  S20. neurodevelopmental disorder* or learning disab* or learning impair* or learning difficult* or learning deficien* or intellectual impair* or intellectual handicap* or intellectual disorder* or intellectual deficien* or handicap* or cognitive impair* or subnormal or mental impair* or mental deficien* or mental disorder*  S21. "cerebral palsy" or "Fragile X syndrome" or "Down syndrome" or "Prader-Willi syndrome" or "Smith-Magenis syndrome" or "Rett syndrome" or "Lesch Nyhan syndrome" or "Angelman Syndrome" or "Cri du Chat Syndrome" or "Cornelia de Lange syndrome" or "Rubinstein-Taybi syndrome" or "DiGeorge syndrome " or "Williams syndrome" or phenylketonuria or "Fetal alcohol spectrum disorder" or "Autis* spectrum disorder" or Autism or "Attention deficit disorder"  S22. S18 OR S19 OR S20 OR S21  S23. S10 AND S14 AND S17 AND S22 |

**Database Host:** Ovid

**Database:** MedLINE

**Date:** 3.2.2022

**Results:** 1,639

**Limits:** English Language; Publication Year: 2000 - 2020

| **Concept 1** | **Concept 2** | **Concept 3** | **Concept 4** | **Concept 5** |
| --- | --- | --- | --- | --- |
| **HRQoL and Subjective Wellbeing** | **Measures** | **Psychometric Properties** | **Adolescence** | **Intellectual Disability** |
| 1. Adolescent/ or Health Status/ or Health/ or Mental Health/  2. "Quality of Life"/  3. Diagnostic Self Evaluation/  4. (subjective wellbeing or objective wellbeing or wellness or wellbeing or well-being or subjective health or or eudaimonic wellbeing or hedonic wellbeing or happiness or life satisfaction or self-concept or social support or mental wellbeing or psychological wellbeing or psychological health or physical health or physical wellbeing or social health)  5. 1 or 2 or 3 or 4 | 6. "Surveys and Questionnaires"/  7. Self Report/  8. (measure* or scale* or indicator* or instrument* or tool* or health outcome* or survey* or questionnaire* or assessment instrument* or assessment* or evaluation)  9. 6 or 7 or 8  10. 5 and 9 | 11. Psychometrics/ 12. (valid* or reliab*)  13. "Reproducibility of Results"  14. 11 or 12 or 13 | 15. Adolescent/  16. (youth* or young people or teen* or child*)  17. 15 or 16 | 18. intellectual disability/ or mental retardation, -linked/  19. neurodevelopmental disorders/ or developmental disabilities/ or intellectual disability/ or learning disabilities/  20. Disabled Persons/  21. (learning impair* or learning difficult* or learning disorder* or learning deficien* or intellectual development disorder or intellectual impair* or intellectual handicap* or intellectual disorder* or intellectual deficien* or handicap* or cognitive impair* or impair* or subnormal or development* delay or mental disab* or mental impair* or mental handicap* or mental deficien* or mental disorder*)  22. ("cerebral palsy" or "Fragile X syndrome" or "Down syndrome" or "Prader-Willi syndrome" or "Smith-Magenis syndrome" or "Rett syndrome" or "Lesch Nyhan syndrome" or "Angelman Syndrome" or "Cri du Chat Syndrome" or "Cornelia de Lange syndrome" or "Rubinstein-Taybi syndrome" or "DiGeorge syndrome " or "Williams syndrome" or phenylketonuria or "Fetal alcohol spectrum disorder" or "Autis* spectrum disorder" or Autism or "Attention deficit disorder").  23. 18 or 19 or 20 or 21 or 22  24. 10 and 14 and 17 and 23 |

**Database Host:** ProQuest

**Database:** ERIC

**Date:** 3.2.2022

**Results:** 450

**Limits:** English Language; Publication Year: 2000 –2020

| **Concept 1** | **Concept 2** | **Concept 3** | **Concept 4** | **Concept 5** |
| --- | --- | --- | --- | --- |
| **HRQoL and Subjective Wellbeing** | **Measures** | **Psychometric Properties** | **Adolescence** | **Intellectual Disability** |
| noft("Quality of Life" OR "Psychological Well-Being" OR "Personal Satisfaction" OR "Wellness" OR "wellbeing" OR "well-being" OR "subjective health" OR "subjective wellbeing" OR "objective wellbeing" OR "eudaimonic wellbeing" OR "hedonic wellbeing" OR "happiness" OR "self-concept" OR "social support" OR "mental wellbeing" OR "psychological health" OR "social health" OR "health related quality of life" OR "Mental Health" OR "health") | AND noft("Self Report*" OR "Scale*" OR "measure*" OR "measure* tool*" OR "survey*" OR "questionnaire*" OR "tool*" OR "instrument*" OR "indicator*" OR "health outcome*" OR "assessment*" OR "evaluation*") | AND noft("psychometric*" OR "reliab*" OR "valid*" OR "reproducibility of results") | AND noft("Adolescen*" OR "youth*" OR "young people" OR "teen*" OR "child*") | AND noft("intellectual disab*" OR "mental retard*" OR "neurodevelopmental disorder*"OR "developmental disab*" OR "learning disab*" OR "disab*" OR "learning impair*" OR "learning difficult*" OR "learning disorder*" OR "learning deficien*" OR "intellectual development disorder*" OR "intellectual impair*" OR "intellectual handicap*" OR "intellectual disorder*" OR "intellectual deficien*" OR "handicap*" OR "mental disab*" OR "cognitive impair*" OR "impair*" OR "subnormal" OR "development* delay" OR "mental disab*" OR "mental impair*" OR "mental handicap*" OR "mental deficien*" OR "mental disorder*" OR "special needs" OR "cerebral palsy" OR "Fragile X syndrome" OR "Down syndrome" OR "Prader-Willi syndrome" OR "Smith-Magenis syndrome" OR "Rett syndrome" OR "Lesch Nyhan syndrome" OR "Angelman Syndrome" OR "Cri du Chat Syndrome" OR "Cornelia de Lange syndrome" OR "Rubinstein-Taybi syndrome" OR "DiGeorge syndrome" OR "Williams syndrome" OR "phenylketonuria" OR "Fetal alcohol spectrum disorder" OR "Autis* spectrum disorder" OR "Autism" OR "Attention deficit disorder") |
